# Archetypes of Service Innovation: Implications for Value Cocreation

**DOI:** 10.1177/1094670517746776

**Published:** 2018-01-01

**Authors:** Anu Helkkula, Christian Kowalkowski, Bård Tronvoll

**Affiliations:** 1 Department of Marketing, CERS—Centre for Relationship Marketing and Service Management, Hanken School of Economics, Helsinki, Finland; 2 Department of Management and Engineering, Linköping University, Linköping, Sweden; 3 Inland Norway University of Applied Sciences Elverum, Norway

**Keywords:** service innovation, value, cocreation, cocreation of value, experience, service systems, service-dominant logic

## Abstract

Service innovation is a key source of competitive differentiation across firms and markets. Despite growing attention from practitioners and academics alike, systematic scholarly inquiry into service innovation’s diverse theoretical foundations has to date been limited. This article explores different approaches to service innovation and proposes a typology of four archetypes, each informed by a distinct theoretical perspective and by different underlying assumptions. Process-based and output-based archetypes focus on value-adding phases and output value, respectively. Experiential and systemic archetypes have attracted less attention but become central for firms seeking to cocreate phenomenologically determined value within the service ecosystem. The article also contributes to service innovation research and practice by bringing together the existing archetypes, which were previously treated separately. Juxtaposing these archetypes and emphasizing value and value cocreation, the article proposes an integrative view of how novel value cocreation can be enhanced in service innovations. Finally, we develop an agenda for future research, encouraging researchers and managers to plan service innovations systematically, deploying each archetype in value cocreation, and combining them within an integrative approach.

Spurred by accelerating technological advances, the service innovation landscape has undergone radical shifts. Service innovation is now seen as the main engine of differentiation and growth, and the body of scholarly research has grown considerably in the past decade (Carlborg, Kindström, and Kowalkowski 2014; Witell et al. 2016). Service innovation has been identified as one of the three strategic priorities for service research (Ostrom et al. 2015). Given the importance of service innovation and the diversity of actors, situations, and contexts, a key priority is to broaden our understanding of innovation and the framework within which it is understood.

Innovation has been addressed from multiple theoretical perspectives, which we refer to here as archetypes. In terms of their salient facets, these archetypes mobilize different underlying assumptions and epistemologies, grounded in a range of academic fields that include marketing (Nijssen et al. 2006), economics (Gallouj and Savona 2009), operations (Edvardsson and Olsson 1996), and strategy (Cusumano, Kahl, and Suarez 2015). With roots in the analysis of technological innovation for manufacturing (Gallouj and Weinstein 1997), service innovation research is characterized by firm centricity and traditionally traces innovation in terms of outputs or processes. The literature has its origins in the distinction between product and process innovation (Abernathy and Townsend 1975; Utterback and Abernathy 1975).

To date, innovation research has focused principally on output and process, the two archetypes of innovation, and the customer has been seen primarily as the recipient of predefined market offerings (Vargo and Lusch 2004). Increasingly, however, researchers have come to consider innovation from a value cocreation perspective. For example, Lusch and Nambisan (2015) cautioned that the product–service distinction should not constrain a broader view of innovation. Similarly, Rubalcaba et al. (2012, p. 697) noted that “innovation is not just a new offering but rather improved customer value cocreation.” On that basis, some recent service research has moved beyond traditional output- and process-based archetypes to a more experiential (phenomenological) and systemic understanding of value creation (Karpen, Bove, and Lukas 2012; Prahalad and Ramaswamy 2003). Drawing on service-dominant (S-D) logic, the increased interest in resource integration as an aspect of value cocreation has also inspired experiential and systemic archetypes of service innovation (Edvardsson and Tronvoll 2013; Helkkula, Kelleher, and Pihlström 2012), emphasizing the social aspects of value cocreation. This broadening of the scope of innovation beyond firm-centered production activities and offerings has in turn generated new knowledge and practical solutions (Lusch and Nambisan 2015; Vargo, Wieland, and Akaka 2015).

In the present study, we consider archetypes as theoretical prototypes. In the literature to date, the various archetypes of innovation and value cocreation have been discussed separately rather than linked. While archetypes may not be isolated in this way in practice, they are of use in characterizing the differing theoretical lenses applied to service innovation (cf. McKelvey 1975; Doty and Glick 1994; Brodie 2014). However, Tether and Howells (2007) warned about narrow, one-dimensional, single-issue research (focusing, for instance, on service offering or process), and service researchers have yet to explicate how the different archetypes of innovation complement one another or create opportunities and challenges for value cocreation.

In support of multiple-lens explanations, Okhuysen and Bonardi (2011) noted the importance of identifying different theoretical archetypes for fuller knowledge integration. To meet this challenge, the present article examines different archetypes of service innovation and their role in value cocreation. In so doing, we contribute to the existing literature in three ways. First, we propose a typology of four theoretical archetypes of service innovation—output based, process based, experiential, and systemic—and clarify their role in value cocreation. Of the four, output-based and process-based archetypes occur most frequently, while experiential and systemic accounts can be characterized as “emerging” archetypes of service innovation. Typologies commonly present “ideal” theoretical archetypes, which in practice do not exist in isolation (Brodie 2014; Doty and Glick 1994; McKelvey 1975). Accordingly, the four archetypes discussed here are not mutually exclusive but what Doty and Glick (1994) refer to as “multiple ideal types,” representing views and forms that *might exist*.

Theoretical typologies are useful for business practice, enabling combination and explication of theoretical assumptions in relation to empirical phenomena; a more developed theoretical apparatus facilitates analysis and eventually prediction. A combined view enables both researchers and managers to analyze service innovation, to diagnose problems in value cocreation, and to implement service innovations that will foster value cocreation. Our second contribution, then, is to address the call for theoretical integration (McInnis 2011). Adopting the perspective of S-D logic, we focus here on understanding service innovation from the perspective of value cocreation. This necessarily depends on the combined view, as no single theoretical archetype alone can capture the complexity of value cocreation in service innovation because of its phenomenological appearances. The combined typology of archetypes in service innovation distinguishes theoretical lenses, facilitating their practical application and so bridging theory and practice in pursuit of novel value cocreation (Brodie et al. 2011; Doty and Glick 1994).

We discuss the theoretical archetypes in relation to two minicase examples, which serve to illustrate how this conceptualization might be applied in empirical settings (Siggelkow 2007). In the first of these, the development of the movie industry reveals shifts in movie watching behaviors over time. In the second case, we consider how TripAdvisor, an interactive platform providing access to a system of tourism and travel resources, provides improved outputs (better trips) based on a new process for navigating, viewing, and choosing (before, during, and after traveling). Users experience and cocreate value with TripAdvisor by mobilizing this platform. Additionally, we refer to other minicases to illustrate how different archetypes inform service innovation.

Our third contribution is to develop a research agenda for service innovation theory development and managerial practice based on the typology of archetypes. As the proposed typology brings together different theories, it expands the scope of existing theories and makes theoretical assumptions more explicit (Brodie 2014; Brodie, Saren, and Pels 2011; Weick 1989).

## Conceptual Foundation: Archetypes of Service Innovation

Traditionally, there are three broad empirically driven views of service innovation, respectively, emphasizing *assimilation*, *demarcation*, or *synthesis* (Coombs and Miles 2000). Assimilation assumes that service activities are generally the same as manufacturing activities. As a consequence, differences between product innovation and service innovation are not acknowledged, and existing theories and models of innovation are considered equally applicable to the service innovation context (e.g., Nijssen et al. 2006; Sicotte and Bourgault 2008). With conceptual roots in the distinction between products and services, the second viewpoint, demarcation, is the antithesis of assimilation (Fisk, Brown, and Bitner 1993). Demarcation emphasizes the unique characteristics of services and the consequent need for specific models and theories to comprehend the nature and dynamics of service innovation (e.g., Edvardsson and Olsson 1996; Gadrey, Gallouj, and Weinstein 1995).

The two schools of thought are unified by their shared conceptual foundation in goods-dominant (G-D) logic, manifesting in an emphasis on individual firms as service producers, customers as service consumers, and studies that privilege product and process innovation (cf. Lusch and Nambisan 2015). As a result, this strand of research has concerned itself primarily with firm-centric attributes (Carlborg, Kindström, and Kowalkowski 2014), focusing either on the service offering itself or on the service process (Ostrom et al. 2015). In a systematic review of service innovation research, Droege, Hildebrand, and Forcada (2009) confirmed this focus on success factors for new service offerings. Whether dealing with goods or with services, then, innovation research has tended to privilege firm-centric production activities and offerings.

In the present study, we draw on the third approach: synthesis, which emphasizes value and characterizes service innovation as multidimensional, with no dominant paradigm (e.g., Michel, Brown, and Gallan 2008; Skålén et al. 2015; Yu and Sangiorgi 2018). On that basis, Carlborg, Kindström, and Kowalkowski (2014) noted the need for a service innovation typology, while Gallouj and Djellal (2010), Gallouj and Savona (2009), and Rubalcaba et al. (2012) have called for the development of an integrative and overarching framework to guide service innovation researchers and practitioners.

The increasing interest in customer experiences, service ecosystems, and resource integration as part of value cocreation—especially in studies informed by S-D logic—has prompted the emergence of experiential and systemic archetypes of service innovation. Along with the established output-based and process-based archetypes, these are needed for a fuller understanding of how firms seek to cocreate value with different actors in the service ecosystem. The shift to S-D logic means that diverging views of service innovation can be unified, offering a more dynamic and holistic lens for exploring value cocreation (Vargo, Wieland, and Akaka 2015). S-D logic offers an overarching view of how value is cocreated, and the emphasis on innovation as synthesis facilitates the integration of diverse views and archetypes (Coombs and Miles 2000). The proposed approach requires the articulation of existing theoretical archetypes and their epistemological grounds. We will first discuss the dominant output- and process-based archetypes of service innovation before turning to the newer experiential and systemic archetypes, which are not colored by traditional product- or process-based assumptions.

With conceptual roots in phenomenology (Husserl 1970), the experiential archetype has been actively discussed in value and value cocreation research informed by S-D logic (Vargo and Lusch 2008) and service logic (Grönroos and Voima 2013). The systemic archetype is grounded in system theory (von Bertalanffy 1971) and industrial network theory (Håkansson et al. 2009). Highlighting contrasts and parallels among the four archetypes, our analysis focuses on the potential of an integrative value-centric view for enhancing value cocreation in service innovation. We go on to articulate an agenda for future research.

### The Output-Based Archetype of Service Innovation

Output is essentially a matter of quantities resulting from the transformation of inputs such as research and development (R&D; Mairesse and Mohnen 2002), and output is what many studies of innovation are actually measuring (Leiponen 2012; Mairesse and Mohnen 2002; Murovec and Prodan 2009; Walker, Jeanes, and Rowlands 2002). When service innovation is viewed as an output, the focus tends to be on attributes, as in critical success factors or performance indicators. Successful service innovation, then, can be conceptualized (in whole or in part) as one or several multivariable constructs related to the outputs of service innovation processes, such as the number of new service offerings (de Brentani and Kleinschmidt 2004; Hull 2004; Sicotte and Bourgault 2008). Output generally corresponds to financial performance or value-in-exchange constructs, reflected in measures of success rate, profitability, or sales impact (e.g., being on schedule and on budget; see Cooper and de Brentani 1991). Storey and Kelly (2001) acknowledged that such analyses should encompass both the individual project level (i.e., success of a new service offering) and the program level (i.e., success of service innovation over time).

In terms of the role of customers in relation to service innovation outputs, we can discern two distinct views. In traditional assessments, the firm serves as an active developer of service innovations while customers are passive adopters—that is, they buy the new service offering and make it profitable for the firm (Sundbo 2001). This view is rooted in the neoclassical economic view in which the separation of production and consumption resonates with the early, unidirectional Schumpeterian innovation model of “the lone entrepreneur bringing innovations to markets” (Laursen and Salter 2006, p. 132). This also relates to Kuznets’ system of national accounts and its use during the World War II to measure productivity and to set production targets for both the military and civilian sectors of the economy (Fogel 2000). However, this view has attracted increasing criticism, especially for its failure to consider the emergence of service innovations (Gallouj and Weinstein 1997)—for instance, internal R&D departments and central development projects are less important for service innovation than for conventional product innovation (Tether 2005). Furthermore, there is evidence that customer participation can have a positive effect on performance and other service innovation output criteria (Edgett 1994), and this is increasingly acknowledged as a key component of service innovation.

To understand how the four theoretical archetypes relate to empirical phenomena, we will begin by examining how firms in the movie industry provide access to movies and how customers watch them. Adopting a longitudinal approach to reveal changes over time (Siggelkow 2007), we can identify three major shifts in movie watching behavior: (1) from watching movies at the cinema to watching movies on television (TV) at home, (2) from watching broadcast movies to watching rented movies at home, and (3) from physically renting movies to renting/watching movies online. According to output- and process-based approaches, such shifts represent technological product innovations. The output-based archetype would focus on service innovations offering new ways of watching movies. Rapid improvements in technology between the 1910s and 1940s saw the opening of thousands of large and often grandiose movie theaters, especially in the United States. When the development of TV allowed movies to be broadcast directly, patterns of movie consumption eventually changed. For people living outside major cities, this change was significant, as their supply was previously very limited. Extensive broadcasting (i.e., more than one channel) allowed customers to watch movies from different genres, countries, and time periods. On an output-based view, this service innovation shift led to increased value creation because customers were able to watch more movies than before. More recently, the option of watching movies seamlessly online on any suitable device has further increased customer flexibility and choice.

Overall, the main contribution of the output-based archetype of service innovation has been characterized service innovation activity in terms of measurable, valuable achievements. This output-based archetype foregrounds how firms innovate service offerings for their customers, even where customers participate in those processes (e.g., lead users). Drawing on traditional innovation research, this archetype is rooted in a product development perspective that equates service innovation with output, defined as the number of new services launched. On this view, service innovation is seen as an economic concept—a reproducible practice that provides a benefit to its developer(s) (Toivonen and Tuominen 2009). The focus on output means that value is seen to be embedded in the service offering, which the customer acquires through a (predefined) value in exchange (Grönroos and Voima 2013) regardless of their level of participation.

### The Process-Based Archetype of Service Innovation

The process-based archetype appears mainly in new service development (NSD) research, which views service as a process (Edvardsson, Gustafsson, and Roos 2005). This perspective emphasizes the architectural elements or phases of the service experience as well as their order (Toivonen, Tuominen, and Brax 2007), which tends to be linear and sequential (Gallouj and Savona 2009). Transformation or change (such as learning) is also emphasized. Explicitly or implicitly, a number of studies have equated service innovation with NSD (e.g., Menor, Tatikonda, and Sampson 2002), and the related research addresses such topics as innovation antecedents (Froehle et al. 2000; Ordanini and Parasuraman 2011), success factors for new services (de Brentani 1995; Martin and Horne 1995; Melton and Hartline 2010), the effect of service innovation activities on firm performance (Mansury and Love 2008; Ordanini and Parasuraman 2011; Storey and Hughes 2013), and customer participation (Alam 2002; Blazevic, Lievens, and Klein 2003; Carbonell, Rodríguez-Escudero, and Pujari 2009). Beyond service innovation, no other service processes are considered.

Analysis of service innovation as a process entails a distinction between a *service innovation process* and a *service process innovation*. While the former pertains to the delivery and success or failure of a new service, the latter refers to the process of service creation; for service delivery innovation, see, for example, Ja-Shen, Hung Tai, and Ya-Hui (2009); for design of service delivery systems, see Zomerdijk and Voss (2010). Voss and Hsuan (2009) suggested that service architecture and modularity could support the decomposition of services into smaller units and more efficient processes. Where the focus is on market offerings, process innovation research tends to be firm centric, emphasizing technology-based process improvements that enhance the performance of existing services. As a well-known proponent of this perspective, Barras (1986) proposed a “reverse product cycle” to conceptualize services in relation to physical goods (see also Ordanini and Parasuraman 2011). More generally, a process-based archetype applies to any change in the service creation process that influences the emergence of value-in-use, including shifts in the roles, competences, skills, practices, or behaviors of a firm’s employees or customers (see also Gallouj and Savona 2009; Gallouj and Weinstein 1997).

Several studies have conceptualized process-based and output-based service innovation as theoretically distinct concepts (e.g., Barras 1986; Edvardsson and Olsson 1996; Menor and Roth 2007; Sirilli and Evangelista 1998; Toivonen and Tuominen 2009). This distinction is useful because these archetypes frame and contribute to the cocreation of value in different ways. In practice, the beginning and end of a process and its relation to output may be difficult to discern, especially in light of the interactive and dynamic characteristics of service (Droege, Hildebrand, and Forcada 2009; Gallouj and Savona 2009; Miles 2008).

Returning to the example of the movie industry, the process-based archetype would focus on changes in the processes of delivering, accessing, and watching the movie. For example, the service became more convenient, as people could watch movies from home and no longer had to drive to the movie theater, book in advance or queue for tickets, or worry about getting good seats. With the advent of movie rentals, customers no longer had to watch the entire movie as broadcast but could decide when to stop the video, take breaks, or watch it again. With the requisite technology, customers can now watch movies anywhere and anytime, which represents a major process service innovation. Where any such shift alters the customer’s role in the value-creation process, Michel, Brown, and Gallan (2008) have suggested that the change constitutes a discontinuous innovation. The role of customer participation in process-based service innovation relates to deliberate and managed user participation at different stages of the service innovation process (Alam 2006). For instance, Oliveira and von Hippel (2011) highlighted how lead users can generate service innovations, and Magnusson, Matthing, and Kristensson (2003) concluded that ordinary users are often more innovative than employees in generating ideas for new service offerings. Chan, Yim, and Lam (2014) noted that customer participation in service processes can be a double-edged sword. While a high level of customer participation may increase customer satisfaction through the creation of economic and relational value, output uncertainty may also increase (Larsson and Bowen 1989) along with job-related stress, so undermining job satisfaction (Chan, Yim, and Lam 2014).

Overall, the main contribution of the process-based archetype is its focus on service innovation activity and time span, in which service innovation is understood as an activity rather than as an output (Toivonen and Tuominen 2009), and the customer is seen to participate in the production process rather than just at the point of output (Grönroos 1998). For these reasons, service processes can vary significantly with different patterns of interdependence and division of work between employees (e.g., front-stage and backstage) as well as different degrees of customer participation. Process innovations can also affect customers’ behavior in various ways either increasing customers’ involvement in value cocreation (e.g., online banking, self-service hotels) or reducing it (e.g., delegation of service activities enabled by automated backstage operations).

### The Experiential Archetype of Service Innovation

The experiential archetype is informed by a phenomenological understanding of experience as individual and subjective. Drawing on Husserlian phenomenology (Husserl 1970), the primary focus of any analysis based on the experiential archetype is the individual service innovation experience and how the customer makes sense of it. In this regard, Smith (2007) referred to the various modes of first-person experience in accessing service innovations, including perception, imagination, thought, emotion, desire, volition, and action. Because they involve individual sensemaking, such experiences are not objective. Helkkula and Holopainen (2011) characterized service innovation as the subjective, individual experience of a service innovation in a social context. Here, subjective experience and sensemaking in the customer’s own social context determine what is considered a service innovation. For example, while one customer may experience the new service process as convenient, exciting, easy, or simply new (Helkkula, Kelleher, and Pihlström. 2012), another may find it difficult or unpleasant.

This conceptualization of service innovation in terms of the experience of customers, employees, or other engaged actors is rarely the starting point for creating new services. However, the archetype has attracted increasing interest precisely because of its focus on customer and user experiences and, in particular, on how customers (users) experience improved value cocreation in service innovations (Rubalcaba et al. 2012). What each individual subjectively experiences as a service innovation requires no merchandizing and need not even be known to the firm. Within the customer’s own social networks, resource integration can also occur without any direct interaction with the service firm (Grönroos and Voima 2013). In some cases, experiential service innovation may even be imaginary (Helkkula, Kelleher, and Pihlström 2012), as for instance when triggered by discussions with other individuals or by indirect communication channels representing a firm’s service, including branding, advertising, news reports, reviews, or electronic word of mouth (Dube and Helkkula 2015; Meyer and Schwager 2007).

In a service context, academic research on the experiential archetype has focused on value (Helkkula, Kelleher, and Pihlström 2012; McColl-Kennedy et al. 2012; Vargo and Lusch 2008). Vargo and Lusch (2008, p. 7) asserted that “value is always uniquely and phenomenologically determined by the beneficiary,” positing that experienced value is uniquely determined by the actor—not only while using the service but also in the wider phenomenological context beyond a given ecosystem. According to Prahalad and Ramaswamy (2003, p. 14), value is cocreated through experience, where value and value cocreation are defined by “the experience of a specific consumer, at a specific point in time and location.” The technology or process is not central but serves merely as a distribution mechanism for service provision, creating no value per se.

As one empirical example of the experiential archetype, consider how movie viewers’ value experiences have changed by virtue of service innovation. Innovative shifts have enabled us to watch movies on TV, as rentals, and subsequently online, anywhere and anytime. In the process, the individual’s experience in their social context has changed accordingly—that is, the experience as a whole can readily migrate from social and collective to domestic and individual. Since the first movie theaters appeared in the early 20th century in Europe and North America, watching movies has become a significant value experience for customers—a potential “wow” experience, in which individuals lose their immediate sense of time and place (Millard 2006). As each individual phenomenologically determines value, service innovations can be seen to have changed the service experience for better or worse, depending on the individual user’s point of view. In general, the main contribution of the experiential archetype of service innovation is its focus on individual improved customer value experiences and value cocreation. All engaged actors experience this value individually and subjectively in their own social context, cocreating value through experience (Rubalcaba et al. 2012).

### The Systemic Archetype of Service Innovation

The systemic archetype is informed by a holistic belief that the whole is more than the sum of the parts (Sheth, Gardner, and Garrett 1988) and that something is lost when focusing on separate parts. In a marketing context, this archetype dates back to the 1960s, influenced by contemporary ideas about social and living systems (Bell 1966; Forrester 1958). While only market-facing resources—that is, as possessed or controlled by the firm—would traditionally have been taken into account when attempting to understand service innovation, a systemic perspective invites consideration of a wider range of resources. On this view, both private-facing resources (possessed or controlled by the individual or customer) and public-facing resources (possessed or controlled by society) become vital elements in service innovation along with the creation or recreation of norms and rules of the system and the broader social context—that is, changes in institutions and institutional arrangements.

In the academic literature on marketing, the systemic archetype emerged from Alderson’s (1965) description of organized behavior systems and has recently attracted renewed interest in the context of service ecosystems (Chandler and Lusch 2015; Spohrer and Maglio 2008; Vargo, Wieland, and Akaka 2015). Vargo and Lusch (2011) argued the need for an ecosystem orientation in order to understand and apply the principles of value cocreation because of their interconnected, dynamic, and varying implications. Rubalcaba et al. (2012) contended that the proper unit of analysis for service innovation research is not the service offering itself but the service ecosystem. This echoes the view of Michel, Brown, and Gallan (2008) who argued that the systemic archetype is a promising line of development for service innovation.

A service ecosystem can be defined as a “relatively self-contained, self-adjusting system[s] of resource-integrating actors connected by shared institutional logics and mutual value creation through service exchange” (Lusch and Vargo 2014, p. 161). On this view, a service ecosystem has two primary roles to enable, facilitate, and guide value cocreation and to foster service innovation (Edvardsson and Tronvoll 2013). In turn, firms must design resource integration mechanisms to link resources, actors, and institutional arrangements and to enable actors to enhance the service innovation. Interactions between actors in the service ecosystem are primarily social encounters (Czepiel, Solomon, and Surprenant 1985), confirming that actors are at the center of every service ecosystem (Tronvoll 2017). Actors are guided by social values and institutional arrangements that determine how resources are to be understood, accessed, used, and integrated in achieving service innovation.

Edvardsson and Tronvoll (2013) described service innovation as involving changes to the structure of the service ecosystem (including resources and institutional arrangements), based either on a new configuration of resources or on a new set of norms and rules, and resulting in new practices that are of value to the actors in a specific context. On this view, service innovation can be seen as embedded in social structures and occurring within social systems, encompassed and shaped by institutional arrangements that enable or inhibit that service innovation (Vargo, Wieland, and Akaka 2015). The systemic archetype broadens the scope of service innovation by emphasizing this totality and incorporating multiple items—other actors in the market, regulations, norms, and rules—in seeking to understand service innovation and its environment.

Again applying the archetype to movie watching, the systemic approach highlights how resource configuration and institutional arrangements have changed across that entire service ecosystem. For example, the mass market breakthrough of TV in the United States in the 1950s created a shift from market-facing resources (movie theaters and machinery and the knowledge and skills needed to operate those facilities) to private-facing resources (the customer’s own home, their TV, and their knowledge of how to operate the TV). The videocassette player subsequently introduced a new entity to the movie service ecosystem in the form of rental shops and chains, creating an additional service encounter from the customer’s perspective. Similarly, the shift from tangible to intangible distribution of movies (what Normann 2001 refers to as “dematerialization of resources”) and the emergence of mobile movie playing devices (such as smartphones) have further disrupted the service ecosystem. Clearly, major service innovations entail profound changes in the service ecosystem or even industry convergence, as new actors (often from new industries) drive change and establish new norms and rules.

The main contribution of the systemic archetype of service innovation is its focus on resource integration by various actors in a service ecosystem. It has been suggested that firms cannot design or create market offerings or develop and manage service ecosystems without connecting with multiple actors in the network that activates the system. Edvardsson, Tronvoll, and Gruber (2011) emphasized that value is cocreated in a social context because service ecosystems are embedded in that context, and customers inevitably evaluate value in use as value in context. The novel value so created must therefore be understood as part of a collective context, embedded in a social system. In practice, service ecosystems are created and recreated by activities and interactions, in which actors integrate and use available resources, guided by the norms and rules of the social context, so enhancing service innovation.

## Discussion and Implications

Typologies based on theory can enhance business practice by supporting new combinations of theoretical assumptions about a given phenomenon, making the theory more explicit. Our study contributes to service innovation theory and practice in three ways. First, we have developed a typology of four existing theoretical archetypes of service innovation—output based, process based, experiential, and systemic—with a focus on value and value cocreation. In demonstrating how the archetypes differ, we also show how this differentiation can add precision to thinking, supporting more comprehensive reasoning about service innovation. Table 1 differentiates the four archetypes of service innovation in terms of three dimensions: key references and characteristics, contributions to value cocreation, and actors’ roles.

**Table 1.** Four Archetypes of Service Innovation and Their Contributions to Value Cocreation.

	Output-Based Archetype	Process-Based Archetype	Experiential Archetype	Systemic Archetype
Foundation of the research approach	Product innovation management	New service development, operations management	Phenomenological (experientially determined) value	Social systems, living systems
Characteristics and focus of service innovation				
Focus	Attributes of the service innovation (e.g., new technology)	The service innovation process; architectural elements (phases) of the customer’s service consumption	Actors’ experiences while using the service and in the wider phenomenological context, extending beyond a specific service innovation	Resource integration by actors engaged in the service ecosystem
Description of service innovation /innovation activity	An offering not previously available to the firm’s customers requiring modifications in the sets of competences applied by the service providers and/or customers	A change in the service creation process requiring modifications in the sets of competences applied by service providers and/or customers	An individual experience of something new or revised	A reconfiguration of resources, actors, and institutional arrangements to enable service innovation
Contributions to value cocreation				
Value conceptualization in service innovation	Value-in-exchange: singular entity at a given point in time that is delivered to the customer and is reproducible; creating outputs (to various extents) with valuable attributes	Value-in-use: accumulated throughout the service process as a stage activity, in which value emerges through transformation or change; the process of applying new ideas or current thinking in fundamentally different ways	Value-in-experience: phenomenological (experientially determined) value: new and valuable experiences that are individually experienced but socially cocreated	Value-in-context: improved viability of the service ecosystem; integration of available resources within the service ecosystem in a specific context
Focus on value cocreation in service innovation	Creating outputs (to various extents) with valuable attributes	The process of applying new ideas or current thinking in fundamentally different ways	Cocreating valuable service experiences through service innovations	Integrating available resources within the service ecosystem in a specific context
Roles of actors in service innovations				
Main actors	Companies that innovate offerings; customers as either passive adopters or active codevelopers	Companies that manage the process; actors who attend the process such as customers	Customers or any other actors in the service innovation phenomenon	Different elements of the system that make resources available for value creation
Service firm	To produce new service offerings	To enable new service processes	To facilitate valuable experiences	To integrate market and other resources in a novel and viable way
Employee(s)	To take the new service to the market; launching, selling, and marketing activities	To divide work between frontline employees interacting with customers and backstage employees producing the service in isolation	To facilitate customers’ experiences of value	To integrate market, private, and other resources in a novel and viable way
Customer(s)	To generate ideas and provide inputs and feedback on new service concepts before they are launched or following launch (“after innovation”)	To more or less actively provide inputs to the service production process	To experience the service innovation phenomenon in their social context	To integrate private resources with other available resources in a novel and viable way
Other relevant actors included in the service innovation	Suppliers and service partners	Service partners and other customers (in individual collective phases)	Other individuals in the social setting	All other actors involved in the service ecosystem
Key references				
	Schumpeter (1934), Cooper and de Brentani (1991), and Storey and Kelly (2001)	Barras (1986), Gallouj and Weinstein (1997), and Michel, Brown, and Gallan (2008)	Vargo and Lusch (2008), Rubalcaba et al. (2012), and Helkkula, Kelleher, and Pihlström (2012)	Edvardsson and Tronvoll (2013) and Vargo, Wieland, and Akaka (2015)

The term *typology* refers to a conceptually derived, interrelated set of ideal types. In practice, the four archetypes in our typology rarely exist in isolation; rather, each represents a unique combination of “first-order” constructs; characteristics, foci, contributions to value cocreation, and actors’ roles (see Table 1). There is a fundamental difference between our typology of service innovation and the many schemes in the literature comprising “classification systems that categorize phenomena into mutually exclusive and exhaustive sets with a series of discrete decision rules”^1^ (Doty and Glick 1994, p. 232). In general, these refer to either-or notions (McKelvey 1975), where a type of service innovation is either in a given category or not (e.g., radical or incremental). Bailey (1994) noted that typologies differ from taxonomies, which classify phenomena on the basis of empirically observable, measurable characteristics.

Based on an extensive literature review, Snyder et al. (2016) identified the four most common classifications describing service innovation: (1) degree of change (radical or incremental innovation), (2) type of change (product or process innovation), (3) perceived newness of the service (new to the market or new to the firm), and (4) means of provision (technology or organization). One example is Gallouj and Weinstein’s (1997) seminal account of service innovation as synthesis. This characteristic-based approach distinguishes between six modes of innovation: radical, incremental, improvement, ad hoc, recombination, and formalization. The differences between these modes essentially concern how and to what extent service characteristics and competences change.

In contrast to such classification systems, our typology encompasses different theoretical perspectives and epistemological assumptions about service innovation. Consistent with Doty and Glick’s (1994) notion of typologies as interrelated sets of ideal types, our approach acknowledges that service innovation projects rarely involve only one archetype. In practice, service innovation projects can accommodate a combination of features from different archetypes and may entail different degrees of change and perceived newness, depending on both archetype and actor. For instance, a service innovation project entailing radical changes in the service ecosystem may yield only incremental changes in the individual customer’s experience.

As illustrated by the movie watching example in the previous section, service innovations are multifaceted phenomena and may encompass multiple archetypes. Archetypes differ in their ontological and epistemological assumptions, prompting different kinds of questions and understandings of service innovation and value cocreation. The minicases in Table 2 illustrate how different archetypes reveal themselves in the same service innovation phenomenon. The aim here is not to identify the most typical or representative cases but to illustrate the plurality of service innovation. For example, in the case of the Engineer Administration System at Toyota Industries, the service innovation initiative focused first on improving the efficiency of internal service processes by investing in a mobile solution for service technicians. However, the project’s success could also be characterized in terms of other archetypes; with improved service quality and availability, customers benefited from a better overall experience; increased utilization of service technicians led to higher output; and automation and connectivity resulted in a reconfigured service ecosystem (Kowalkowski and Ulaga 2017).

**Table 2.** Examples of Service Innovation by Archetype.

Service Innovation	Description	Output-Based Archetype	Process-Based Archetype	Experiential Archetype	Systemic Archetype
TripAdvisor: open, online travel site	TripAdvisor is the world’s largest online travel site, offering advice from other travelers, travel choices, and links for booking service	TripAdvisor offers customers an interactive platform of tourism and traveling. As the innovation activity is presented in terms of measurable, valuable aspects, the focus is on the design and technical finesse of the service	TripAdvisor’s customers navigate an improved process that enables them to make more informed decisions and reduce transaction costs and perceived risks	TripAdvisor facilitates customers’ experiences of managing tourism and traveling, a sense of empowerment, and information sharing with others about various travel destinations	TripAdvisor directs customers to centralized online booking of hotels, restaurants, and guided tours, and customers can access other users’ reviews and content
Tide Dry Cleaners: dry cleaning services with multiple options	Tide Dry Cleaners is Procter & Gamble’s innovative franchising concept in the U.S. dry cleaning market	Tide Dry Cleaners offers efficient, high-quality, 24-hr dry cleaning services with multiple additional options such as alterations, leather conditioning, and wedding dress preservation	The customer process includes convenient curbside assistance, such as drive-through valet drop-off and pickup; 24-hr access is provided through lockers, drop boxes, and kiosks. Some stores also offer home and business delivery. Each garment is inspected at seven points in the cleaning process	Tide Dry Cleaners offers customers consistent quality and a convenient experience in airy, fresh locations, with well-trained and customer-oriented employees	The service builds on a franchising model with a network of local franchisees. The interaction between franchisees and the management team is an important element of the concept, as are partnerships with actors such as Green Earth Cleaning
Mobisol: electricity services to off-grid rural households in East Africa based on a photovoltaic system and software	Customers pay a service fee for a solar power system (controller, battery, and panel); selected appliances (e.g., TV, phone charging kit, torch, radio, hair clipper); and maintenance	Rural households can get electricity from an off-grid, rent-to-own solar power system, with various service levels and packages	Mobisol provides free installation and a support hotline, relying on remote monitoring and analytics to manage and charge for the service. Customers use a convenient mobile payment process	Customers experience reliable electricity-based household services (replacing kerosene lamps and firewood with LED torches and cooking stove); entertainment (TV, radio, and stereo); and business (multicharger, hair clipper, iron, etc.), enhancing quality of life and helping to create new jobs	Mobisol relies on sales agents, local service technicians, software engineers, and mobile payment operators. The need for high-quality products requires tight channel control of device manufacturers in China. Issues of classification and taxation require government relationships
OCTOPUS: ABB Marine’s vessel management and advisory system	ABB Marine equips vessels and fleets with integrated marine solutions (software and sensors) for optimal reliability, flexibility, and energy efficiency	The OCTOPUS system provides customers with a wide range of performance management services for energy efficiency optimization and safer voyages	Using weather and loading data to plot the safest and most efficient route, ABB Marine enables fleet managers and ship’s officers to plan and navigate more efficiently and effectively, backed by 24/7 remote support	By installing a comprehensive system for proactive service operations, ABB Marine offers its customers an experience of smoother operation and better control of their fleets through a single interface	ABB Marine can remotely monitor customers’ fleets and provide a proactive service. ABB’s technology platform orchestrates a network of engaged actors in what is a complex service ecosystem
SOIL: container-based sanitation service in Haiti	Sanitation is a major problem in densely populated slums at risk of flooding. SOIL offers affordable sanitation services, with toilet rental at US$3–5/month. (since the 2010 cholera outbreak, people have become increasingly aware of the health impacts of sanitation)	SOIL’s simple, eco-friendly composting toilets can be used in the home rather than having to rely on public toilets or defecating outdoors	SOIL offers a safe and convenient sanitation process with consistent collection and replacement of containers. This high-quality sanitary service reduces exposure to health risks	SOIL facilitates a dignified and reliable sanitation experience to a vulnerable urban community	SOIL collects and replaces the locally produced containers to ensure proper handling of waste. SOIL is funded by private and institutional donors. As part of a global expert network on sanitation (e.g., Kenya and Peru), SOIL collaborates with global institutions.
Uber: technology platform connecting driver partners and riders	Uber is an on-demand transportation service that has revolutionized the taxi industry across the world	Uber offers an app-based option for conventional taxi services at lower prices using fleet management of private drivers and cars	To use the Uber app, customers simply tap the smartphone to specify their pickup location and choose the service. Customers use their Uber account to pay for the ride in advance. The Uber app uses location services to identify available cars that receive the customer request	Uber facilitates customers’ traveling experiences by offering an inexpensive cab to arrive in the minimum possible time	Uber is an app-based service ecosystem platform for drivers and customers. Uber builds a network of people who are willing to become part-time or full-time taxi drivers
KidZania: an indoor amusement park for kids	KidZania of Mexico is a child-sized interactive city combining inspiration, fun, and learning through realistic role-play	KidZania is a novel constellation of equipment, organized as territorial space offering fun time for children	Children learn how working life functions through a role-play process that includes assignments. Parents can keep track of their children remotely as they perform their tasks	KidZania offers children a taste of the real world, offering opportunities for adult-like experiences in such roles as firefighter, construction worker, police officer, and fashion designer, among others	KidZania plays a key role in bringing brands from different industries, industry organizations, and local and regional authorities together in a “constellation of fun.” Partners include American Airlines, Coca Cola, McDonald’s, and Procter & Gamble
Eataly: a high-end Italian food hall	Eataly is an in-store and online retailer of a wide variety of Italian foods and beverages at counters and restaurants	Eataly offers a broad range of high-quality Italian food and beverages in a pleasant environment	Eataly offers customers an easy shopping process for high-quality Italian food and beverages	The Eataly customer experience combines excellent Mediterranean cuisine with Italian food and wine culture and history	Eataly has become known as an eco-friendly in-store and online service ecosystem for well-known Italian food and beverage brands
EASY: a mobile solution for service technicians at Toyota Industries	EASY was the first advanced mobile solution for service technicians in the material handling division, providing support in the repair and maintenance of forklifts	New hardware and software integrated with the firm’s enterprise resource planning system enables more consistent service output quality and higher utilization of service employees. EASY is also an essential component of more recent advanced service offerings such as fleet management solutions	More efficient and effective service processes with automated back-office operations reduce administration for customers, with improved invoicing lead time and replenishment of parts. Service technicians have instant access to service orders, spare parts information, and product usage data	A revised experience for customers and users (i.e., technicians); customers experience Toyota’s material handling service business as more professional and tech savvy. While overall reception exceeded expectations, some (older) employees expressed disapproval of the new ways of working	Collaboration with software and hardware suppliers for a reconfigured, cutting-edge service ecosystem. Close cooperation was needed between regional headquarters and local sales companies for testing, implementing, and upgrading processes and systems. Limitations such as mobile data connectivity initially constrained the service

*Note.* SOIL = Sustainable Organic Integrated Livelihoods; EASY = Engineer Administration System; TV = television.

### A Combined Value-Centric View of Service Innovation

Our second contribution is to propose a combined value-centric view of service innovation that exploits the strengths of each of the above archetypes and overcomes their limitations by accommodating complexity and addressing value cocreation among different actors. The originality of this contribution is that it offers a new way of integrating the archetypes within an overarching view, linking them through an account of innovation as synthesis and conceptualizing service innovation in terms of value cocreation. McInnis (2011) has suggested that this type of reconceptualization makes an important theoretical contribution. Previous research has examined different types of service innovation in isolation rather than in parallel (cf. Okhuysen and Bonardi 2011), but in practice, firms face multiple cocreation challenges that can best be addressed by drawing on different archetypes. Typological and middle-range theory (Brodie 2014) can yield new theoretical perspectives on a given phenomenon (Corley and Gioia 2011); as Witell et al. (2016) pointed out, “sharing an overall view of service innovation enables theory building and research to better operationalize service innovation in further empirical studies” (p. 2870).

The combined value-centric view invites researchers and managers to apply the archetypes in combination to improve cocreation of value for various actors or to enhance the overall viability of the ecosystem. Service innovations involve new types of resource configuration and related changes in institutions and institutional arrangements, which are dynamic and change in response to events (Edvardsson and Tronvoll 2013). While experiential and systemic archetypes view value cocreation as phenomenologically determined by engaged actors in a given social system, output- and process-based archetypes focus on effective outputs and processes. The well-known metaphor of a group of blindmen studying different parts of an elephant is helpful in describing the combinatory nature of a new theoretical perspective. In analyzing the elephant, each man touches a different part of the animal such as the tail or the ear; a holistic view emerges only after the different analyses are compared and combined. In the same vein, we propose a value-centric view that combines the four archetypes to elucidate service innovation as improved cocreation of value. Any analysis always depends on an ontological and epistemological position, and Okhuysen and Bonardi (2011, p. 10) emphasized that when combining theories, authors should “clearly identify and state their own ontological position and use it as a driver.” In the combined value-centric view, we draw on recent work in S-D logic on value and value cocreation, and on service innovation as cocreation of value (Rubalcaba et al. 2012; Vargo and Lusch 2008). This means that even where we employ all four archetypes, they will not share an “equal footing” (cf. Okhuysen and Bonardi 2011) within the value-centric view.

**Figure 1.**
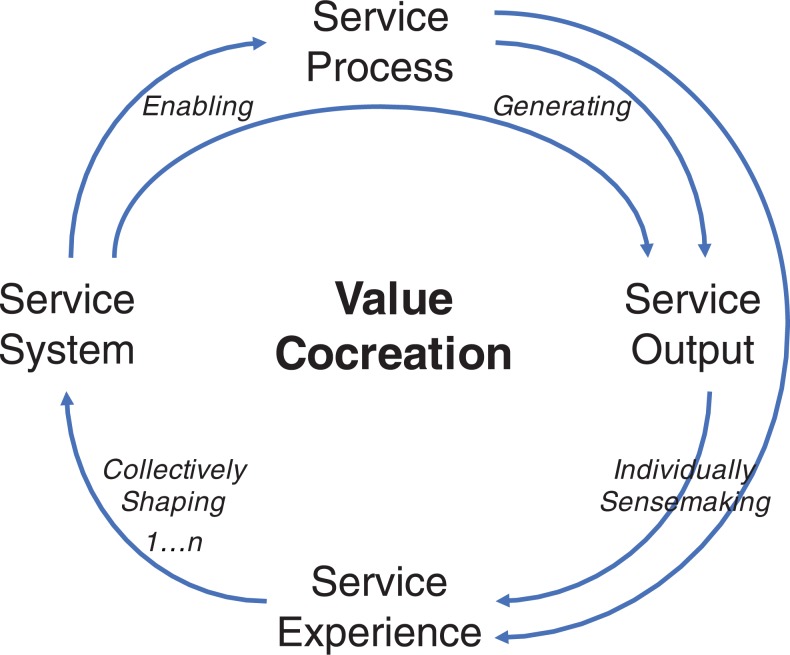
The narrative of value-centric service innovation.

To apply the value-centric view to an empirical business phenomenon, it is useful to revisit the movie watching example to demonstrate how a focus on one specific archetype may neglect other aspects of value cocreation. For each of the major shifts in movie watching behavior, value cocreation was influenced by all four archetypes as illustrated in Figure 1. First, the process-based archetype focuses on how technological innovations change the process of delivering, accessing, and watching the movie. Such changes generate a service output offering new ways of watching movies, such as seamless movie consumption across technical platforms. Through individual sensemaking, service process and output influence an individual’s experience in their social context. Collectively, the service experience influences institutional arrangements across the service ecosystem, which may alter established norms and rules. Reconfigurations of the service ecosystem (such as a shift from market-facing to private-facing resources) in turn facilitate new or improved service processes and further outputs.

By way of illustration and simplification, we consider TripAdvisor as an example of how the value-centric view combines and extends archetypes (see also Table 2). This complements the movie watching case by showing a single firm (the world’s largest online travel site) offering advice from other travelers, travel choices, and links for booking services (TripAdvisor 2017). Seen in terms of the output-based archetype, service innovation means innovating a new benefit or a solution. This is a common approach to service innovation but lacks the holistic view of value cocreation. Instead, the process-based approach focuses on improving the provider’s processes and facilitating service delivery, often driven by cost efficiencies for the provider, customer, or both. TripAdvisor’s customers can make more informed decisions by navigating an improved interface that offers advice from other travelers, travel choices, and links for booking services.

Again, designing a service using only one archetype may lead to improved efficiency by enhancing service delivery mechanisms, but it also risks neglecting the core service and customer experience. The experiential archetype focuses on improving customer value experiences and value cocreation in a social context, but this may depend on a functioning service process. TripAdvisor’s platform and process facilitate experience and content sharing (e.g., reviews and images) related to various travel destinations. A systemic perspective foregrounds service ecosystems and how innovation can better cocreate value. TripAdvisor directs customers to centralized online booking of hotels, restaurants, and guided tours, and customers can access other users’ reviews and critiques. In this way, the systemic archetype is important for value cocreation, as firms and customers do not operate in isolation. Overall, customers become more empowered and can hope to reduce their transaction costs and risks (see also Sigala 2017).

Adopting a combined, value-centric view, service innovation can be understood in terms of new ways of cocreating value. With reference to Rubalcaba et al.’s (2012) view that service innovation is improved value cocreation involving customers, we contend that the combined value-centric view enables researchers and managers to see the strengths and limitations of each archetype for value cocreation.

### Research Agenda for Theory Building and Managerial Practice

Based on the combined value-centric view of theoretical archetypes to value cocreation in service innovations, our third contribution is a research agenda that encourages the further development of middle-range theory. Corley and Gioia (2011) call on both academics and organizations to apply *prescience* in theorizing, defining this as “what we need to know” to advance theory development and managerial practice. Among recent studies emphasizing the need to develop research on value-based service innovation, Rubalcaba et al. (2012) referred to a cocreation logic that prioritizes improved customer value rather than the new offering (see also Michel, Brown, and Gallan 2008; Ordanini and Parasuraman 2011). However, despite calls for an overarching, multidimensional view of service innovation, such studies remain rare.

The proposed research agenda for archetype-specific and value-centric research in service innovation raises questions about existing efforts to advance theory development and managerial practice (see Table 3) and serves to clarify the nature of service innovation. This research agenda also has practical utility—that is, it relates to real-world phenomena—which Corley and Gioia’s (2011) claim is an essential aspect of theoretical development. For present purposes, practical utility refers to the potential to operationalize and test the value-centric view in further empirical studies. As most of the existing work has been conceptual, we would argue the need for further empirical research to augment existing evidence.

**Table 3.** Research Agenda and Managerial Implications for Archetype-Specific and Value-Centric Research in Service Innovation.

Output-Based Archetype	
Research Topic: Analysis of Results or Effects Following Service Innovation Output	Key Managerial Issues
How do service innovations affect market outputs? Whose perspective should determine the output, and which service ecosystem is involved? What methods and tools can be used to analyze the results or effects of a service innovation output? What types of resource are needed to achieve optimal output?	To review the novelty of the service (for the market and the firm) and how it can be successfully introduced to the market To understand what new service offerings will enable customers to perform new activities To consider whether the new service complements or cannibalizes the existing portfolio To recognize the multidimensionality of service innovation when evaluating the financial performance, including (where relevant) both short-term and long-term effects
Process-Based Archetype	
Research Topic: Analysis of Management of Service Processes for Service Innovation	Key Managerial Issues
How can service processes and infrastructure be redesigned over time? What are the key aspects of value cocreation in a service innovation process in managing and allocating resources for all relevant actors? In what ways could service innovation processes be more systematic and focused on customer and employee resources? How can service innovation processes support value cocreation?	To review the internal and external effects of new, redesigned, standardized, automated and/or eliminated service processes To manage the customer’s role in cocreation; to understand and influence customers’ disposition to participate in service processes To ensure the alignment of processes with service partners and other key players (competences, commitment, etc.) To train and motivate the actors involved
Experiential Archetype	
Research Topic: Elaboration of Customers’ and Other Actors’ Value Experiences of Service Innovation	Key Managerial Issues
How do customers and other relevant actors experience service innovations? How can companies facilitate valuable service innovation experiences? How to balance value creation for all relevant actors, given their individual and subjective experiences of value? How can sensemaking be added to service innovation, based on what customers and other actors experience as valuable in their lived business or private lives, and what they are willing to cocreate?	To understand (and potentially influence) the various social contexts in which the service will be experienced To strengthen emotional ties with customers by empowering them To understand staff experiences when managing the new service To understand possible negative experiences among groups of customers and other actors, and how to deal with these
Systemic Archetype	
Research Topic: Understanding Where to Focus the Service Innovation Process in the Service Ecosystem	Key Managerial Issues
How does service innovation change customers’ and other actors’ roles in cocreation? How can changing cocreation roles trigger service innovation? How can service innovation be triggered by changing cocreation and resource integration roles and analyzing customers’ social networks as they integrate resources? How should system viability and actors’ systemic situation be viewed to derive innovative ideas from their interpretation of the situation and their associated capability to cocreate value?	To identify key actors who influence the success of the innovation in a service ecosystem To acquire the requisite network orchestration skills for mobilizing all relevant actors To understand the importance of changing norms and rules To take account of the role of mesofactor and macrofactor such as institutions, regulations, and policies
Combined Value-Centric View	
Research Topic: Portrait of Service Innovation as Improved Cocreation of Value	Key Managerial Issues
What are the benefits and challenges of combining different archetypes in service innovation? How can service innovation projects be managed to exploit the benefits and avoid the challenges embedded in the various archetypes? How can actors improve cocreation of value for themselves? How can the value-centric view be used to improve the viability of the service ecosystem as a whole?	To manage the interplay between the different archetypes in practical service innovation projects To consider the relevance of service innovation archetypes in each case of service innovation To recognize that successful service innovation is multifaceted—for example, that the viability of the overall system may increase and that customers will adopt the new norms and rules even if the original supplier fails to make sufficient profit To acknowledge that success can mean different things to different actors To provide benefits to the organization, customers, other stakeholders, or society as a whole, with due regard to possible negative consequences and outcomes

### Implications for Practice

A better understanding of combinations of archetypes and their application to different areas of business is also likely to identify new avenues for service innovation practice. In this regard, Table 3 lists key issues for decision makers with responsibility for service innovation strategy and implementation. For example, the output-based archetype (new service offerings) becomes relevant when an organization needs to express its performance in terms of service offerings and quantities such as units and numbers. Output-based measures are important both for the organization itself and for most of the engaged actors (including customers, partners, and authorities) for budgetary planning and reporting, statistical, and taxation purposes. The process-based archetype is important for value cocreation where the goal is to create a new process or to improve the quality, efficiency, or effectiveness of an existing service process.

The experiential archetype of service innovation becomes important when practitioners look to cocreate novel value with customers, helping to identify individual experiences of value and customer motivation to adapt and use a specific service innovation. Clearly, customers tend to be more willing to pay for something they experience as valuable. Finally, the systemic archetype focuses on how available resources are integrated by engaged actors when cocreating novel value, how the business model will be restructured, and where service innovations can occur in the service ecosystem. Michel, Brown, and Gallan (2008) noted that a service innovation improves at least one of the customer’s cocreation roles (using, buying, or paying). They also emphasized the importance of innovative value cocreation with the customer, given that cocreation involves customers in the service innovation process as active integrators who combine diverse resources. A real service innovation shift may depend on this systemic archetype to change the roles of resources and actors, how those resources are integrated, and how value is cocreated. The social constructionist approach to service innovation is closely associated with the systemic archetype, emphasizing the roles of different actors in cocreation (Edvardsson, Tronvoll, and Gruber 2011). Although key actors and their roles may change with service innovation shifts, resources are always integrated within a service.

As firms must balance their service innovation efforts (Witell et al. 2016), such projects can more effectively be managed by combining different archetypes to exploit their benefits and avoid any challenges they may entail. This will in turn influence the focal issues associated with that service innovation, and any firm pursuing service innovation should consider all four archetypes and the interdependence between them. Here, attention should also be paid to any unintended and undesirable consequences of service innovation, which are rarely studied (Sveiby, Gripenberg, and Segercrantz 2012). For example, reverting for a moment to the movie watching example, some customers may find that an innovation shift, such as online movie streaming, does not create the same wow experience as watching a movie in a movie theater. Furthermore, as digitization enables new service innovations (e.g., ABB, Mobisol, Toyota, TripAdvisor, and Uber; see Table 2), managers should avoid becoming too immersed in technical issues (a product-centric approach) at the expense of a fuller understanding of potential customers and their value-creation processes. As prior research has shown (e.g., Perks et al. 2017), failure to understand or articulate the complexity and intangibility of novel service opportunities may impede an innovation’s development and launch.

Based on a synthesis of existing archetypes from a value cocreation perspective, we present a four-stage process to help managers to pursue a value-centric approach to service innovation (see Table 4). In line with S-D logic, Rubalcaba et al. (2012) suggested that the logic of cocreation should focus primarily on the cocreation of value rather than on the service offering or output. In developing a service innovation project, the four-stage process begins with identification of the four archetypes, analyzing their potential for improved value co-creation with customers and other relevant actors. Based on that analysis, a decision can be made about the optimal combination of archetypes and approaches to value for the project in question. Evaluation of the different archetypes’ utility remains relevant throughout the project.

**Table 4.** Four-Stage Process for Managers Pursuing a Value-Centric Approach to Service Innovation.

Stage	Description
Identification	All four archetypes need to be considered for more comprehensive identification of new technology- and market-based opportunities for value cocreation with the various actors in a service ecosystem (value-centric approach)
Analysis	For each case of service innovation, the relevance, interplay, and integration of archetypes needs to be understood and analyzed. Output-based archetype: creating novel outputs with valuable attributes (value-in-exchange) Process-based archetype: applying new ideas or current thinking in fundamentally different ways throughout the service process (value-in-use) Experiential archetype: cocreating valuable service experiences for all involved actors (value-in-experience) Systemic archetype: integrating resources within the service ecosystem (value-in-context)
Action	Adopting a combined value-centric view, deployment of an applicable set of resources and capabilities facilitates better value cocreation with customers and other key actors, from start-up to scale-up
Evaluation	Different combinations of archetypes must be evaluated, along with their suitability for value cocreation with customers and other stakeholders. Evaluation is ongoing before, during, and after each service innovation project

The value-centric approach can be applied to any actor’s perspective. However, by implication, a practical service innovation project should focus on customer value cocreation. In particular, it is important to understand how individual customers might experience the value and value cocreation of (a) a given service ecosystem (value-in-context), (b) using that service (value-in-use), or (c) the service output (value-in-exchange). Value cocreation should also be investigated from a management perspective: how to facilitate value experiences for customers in the given service ecosystem, how to manage the service process and resources where the network that activates the service ecosystem involves multiple actors, and what marketable service offering output (e.g., face-to-face service) would yield value-in-exchange. The typology of the four existing archetypes, and especially the proposed value-centric view, provides a foundation for the use of S-D logic to improve value cocreation through service innovation.

## Conclusions

In addressing the importance of further research exploring value cocreation in service innovation, our process of theorizing involves comparison through both differentiation and integration (McInnis 2011; Weick 1995). First, we have proposed a typology by differentiating known archetypes in service innovation research. This delineates a conceptual domain that combines prevailing output- and process-based archetypes with emerging experiential and systemic archetypes, elucidating different possibilities and challenges for the cocreation of value in service innovation research. Our typology and the characteristics of each archetype in Table 1 help to alleviate the semantic, conceptual, and epistemological confusion that has characterized the service innovation literature. By abstracting service innovation modes as four archetypes expressing different ontological and epistemological positions, we have sought to generalize and illuminate the core features of service innovation.

Second, by integrating the four archetypes’ differing contributions to value, we have proposed a value-centric view that offers an overarching view of value cocreation. Here, we draw on Prahalad and Ramaswamy (2003, p. 12) who have argued that “The next practices of innovation must shift the focus away from products and services and onto experience environments—supported by a network of companies and customer communities—to co-create unique value for individual customers.” On this view, value resides in the experience of cocreation in service ecosystems, supported by service processes and implemented as outputs. Grounded in S-D logic, this value-centric view of service innovation emphasizes actors’ value experiences and resource integration in value cocreation. This view complements (and transcends) archetypes of service innovation that are firm centric and product, process, or technology centered. The minicase examples illustrate how a successful firm attends to multiple issues: service offering, process, and service ecosystem, as well as how different actors experience value on using the service innovation. Finally, we outline a research agenda to address the multifaceted nature of service innovation based on a theory-based, value-centric view that can inform service innovation management and practice for the benefit of firms, customers, and other stakeholders. According to this value-centric view, service innovation is not reducible to output, process, experience, or system; instead, improved value cocreation in service innovation rests on a combination of these archetypes.

## Note

1. In practice, the three terms—typology, classification scheme, and taxonomy—are frequently used interchangeably.
